# The Impact of Alcohol-Related Presentations to Emergency Departments on Days with a Public Holiday or Sporting Event: A Retrospective Cohort Study

**DOI:** 10.1017/S1049023X24000232

**Published:** 2024-06

**Authors:** Stephanie Rae Hagan, Julia Crilly, Jamie Ranse

**Affiliations:** 1. School of Nursing and Midwifery, Griffith University, Gold Coast, Queensland, Australia; 2. Department of Emergency Medicine, Gold Coast Health, Gold Coast, Queensland, Australia; 3. Menzies Health Institute Queensland, Griffith University, Gold Coast, Queensland, Australia

**Keywords:** cohort study, emergency nursing, evidence-based emergency medicine, holidays, sports

## Abstract

**Introduction:**

The consumption of alcohol within the Australian community continues to rise, impacting care delivery in already over-burdened emergency departments (EDs).

**Study Objective:**

This study aimed to examine the impact of alcohol-related presentations (ARPs) to EDs on days with a public holiday or sporting event.

**Methods:**

A retrospective cohort study was undertaken using routinely collected health data pertaining to patient presentations diagnosed with an alcohol-related disorder (ICD-10-AM code F10) to two EDs in Queensland, Australia from January 1, 2016 – December 31, 2020. Descriptive and inferential statistics were used to describe and compare ARPs on event days versus non-event days and uncomplicated versus other ARPs on event days only.

**Results:**

Of all 5,792 ARPs, nine percent (n = 529) occurred on public holidays or sporting event days. When compared by day type, type of presentation, mode of arrival, and day of week differed between event and non-event days. On event days, uncomplicated ARPs differed to other ARPs, with uncomplicated ARPs being younger, having shorter median length-of-stay (LOS), and less likely to be admitted to hospital.

**Conclusions:**

In this multi-site study, public holidays and sporting events had a noteworthy impact on ARPs to EDs. Focused refinement on the clinical management of uncomplicated ARPs is warranted to inform future resource allocation, including on event days.

## Introduction

Alcohol is a central nervous system depressant which alters communication between the brain and body, causing symptoms of poor concentration, slower reflexes, and impaired decision making.^1,2^ Excessive alcohol consumption can result in addiction, dependency, and misuse, cascading an array of social stressors^1^ such as relationship strain and subsequent breakdown, loss of job and associated financial difficulties, and suicide.^3,4^ In Australia, the consumption of alcohol continues to rise with one in four people (25.8%) exceeding the recommended guideline of consumption in 2021.^5^ Furthermore, the alcohol-induced death rate is the highest in 10 years at 4,813 in 2019.^6^ This impact within the Australian community has a flow-on effect to the health care system, including the emergency department (ED).

From 2019-2020 and 2020-2021 (two years), there were 160,919 hospitalizations across Australia with a principal primary diagnosis of alcohol intoxication.^6^ The number of alcohol-related presentations (ARPs) is likely under-reported, as Australian EDs are not mandated to screen for or collect alcohol-related data.^7^ Additionally, alcohol may be classified as secondary to another primary diagnosis, such as trauma, and therefore further under-reported. Alcohol-related presentations to EDs can impact staff job satisfaction,^7,8^ patient flow and waiting times,^7^ and places staff at increased risk of workplace violence.^8^

Alcohol-related presentations to EDs can be dichotomized in terms of being “uncomplicated” and “complicated.” Literature suggests an uncomplicated ARP is non-life-threatening, where the patient experiences mild symptoms of intoxication such as: nausea/vomiting, headache, fatigue, dehydration, and body tremors,^1^ and there are no other obvious injuries, overdose, or psychiatric conditions that might require additional investigation or treatment.^9^ Conversely, a complicated ARP is reported to have potentially life-threatening consequences^2^ with moderate to severe symptoms including chronic alcohol-related conditions, respiratory depression requiring airway support, disorientation, confusion, and/or seizures.^1,2^ Other complicated ARPs are those with additional injuries associated to trauma, falls, or motor vehicle accidents, and where pre-existing comorbidities have been exacerbated by the consumption of alcohol, including diabetes, liver failure, and heart failure.^1,10^ Uncomplicated and complicated ARPs have different management pathways, resource requirements, and follow-up requirements.

Mass-gathering events, social and cultural events, and sporting events are increasing in frequency.^11–13^ The consumption of alcohol at these events heightens behaviors due to social excitement and increases the likelihood for self-harm,^14^ violence, disorderly behavior, injuries, and driving under the influence.^15^ As a result, this can increase the likelihood of an ARP to ED.^16^ With binge drinking attributed to 75% of all alcohol consumed,^12,17^ cause for concern continues, particularly on days with a public holiday or sporting event where excessive alcohol consumption^15,18^ and binge drinking behaviors are not uncommon.^17^

The aim of this research was to examine the impact of ARPs to EDs on days with a public holiday or sporting event. This research was guided by the question: What are the differences in patient demographics, presentation characteristics, and health service outcomes for ARPs to EDs on a day with a public holiday or sporting event versus all other days?

## Methods

### Design and Setting

This was a retrospective cohort study undertaken across two public hospitals located in Queensland, Australia. One hospital is a major tertiary and Level 1 trauma center, and the other is a major regional hospital. The two EDs had a combined total of approximately 190,000 ED presentations in 2023.^19^

### Population and Sample

The population included all presentations made to the two EDs from January 1, 2016 through December 31, 2020. The sample of this population included those who had an International Statistical Classification of Diseases and Related Health Problems, Tenth Revision, Australian Modification (ICD-10-AM) code of F10 (ie, alcohol-related disorder) as their primary ED discharge diagnosis.

### Data Collection

Routinely collected data from ED electronic databases Emergency Department Information System (EDIS) and then FirstNet (a module of Queensland Health’s integrated electronic medical record system) were extracted and provided to researchers in Excel (Microsoft Corp.; Redmond, Washington USA) format by a member of the hospital’s Health Informatics Directorate team. Data extracted and used included variables relating to patient demographic characteristics (ie, age, sex, and Aboriginal and/or Torres Strait Islander status); ED clinical characteristics (ie, mode of arrival, presenting problem(s), Australasian Triage Scale [ATS] category, date and time of arrival and discharge, and ED diagnosis); and health service outcomes (ie, discharge disposition from ED and ED length-of-stay [LOS]).

### Data Cleaning and Coding

Cleaning of retrieved data involved checking for outliers. For variables where sex was “unidentified,” age was entered as ≥100 years (a practice often used for “unknown” patients), and LOS in the ED was ≤0 minutes or ≥6,000 minutes (based on assessing the LOS frequency distribution and previous research),^20^ these specific data points were treated as “missing data” for the analysis of these variables.

Four variables required recoding: (1) day of arrival; (2) type of presentation; (3) “frequent presenters;” and (4) mode of arrival. Day of arrival was recoded to “public holiday” or “sporting event” or “other.” If a presentation occurred on a public holiday or sporting event, these were then termed an “event day.” Presentations made on all other days were termed as a “non-event day.”

The type of presentation was recoded into “uncomplicated” ARPs and “other” ARPs. The process of recoding ARPs involved consultation with expert ED clinician researchers comprising three doctors and six nurses. All researchers involved in this process retrospectively determined which “presenting problems” of people with an F10 ICD-10-AM code constituted an uncomplicated ARP. Nine presenting problems were classified as “uncomplicated.” Where 100% agreement between researchers could not be met, these presenting problems were considered to be “other” ARPs. Subsequently, a total of 122 presenting problems were classified as “other” ARPs (Supplementary Table 1 and Table 2; available online only).

**Table 1.** Patient Demographics of Alcohol-Related Presentations to EDs on Event Days versus Non-Event Days

	Event Day^a^ (n = 529)	Non-Event Day (n = 5,263)	Total (n = 5,792)	P Value
**Patient Demographics**				
*Age,* M (IQR)^b^	36 (22-50)	36 (21-51)	36 (21-51)	.819
*Sex,* n (%)^b^				.806
Male	288 (54.4)	2893 (54.9)	3181 (54.9)	
Female	241 (45.5)	2367 (44.9)	2608 (45.0)	
*First Nation,* n (%)				.815
Aboriginal and/or Torres-Strait-Islander	13 (2.4)	140 (2.6)	153 (2.6)	
Not Indigenous	503 (95.0)	4971 (94.4)	5474 (94.5)	
Not Stated/Unknown	13 (2.4)	152 (2.8)	165 (2.8)	

Abbreviation: ED, emergency department; IQR, interquartile range.

a Event days included public holidays, sporting events, and the day prior and post certain public holidays and sporting events. For further specific dates, see Supplementary Table 3 and Table 4.

b Analysis excludes patient presentations where age ≥100 years and sex were unidentified.

**Table 2.** Presentation Characteristics of Alcohol-Related Presentations to EDs on Event Days versus Non-Event Days

	Event Day^a^ (n = 529)	Non-Event Day (n = 5,263)	Total (n = 5,792)	P Value
**Presentation Characteristics**				
*Type of Presentation*, n (%)				.011^b^
Uncomplicated	123 (23.2)	984 (18.6)	1107 (19.1)	
Other	406 (76.7)	4279 (81.3)	4685 (80.8)	
*Mode of Arrival*, n (%)				<.001^b^
BIBA	451 (85.3)	4155 (79.0)	4606 (79.5)	
WI	48 (9.1)	855 (16.2)	903 (15.6)	
BIBP	30 (5.7)	252 (4.8)	282 (4.9)	
*ATS*, n (%)				.743
1, 2	82 (15.5)	795 (15.1)	877 (15.1)	
3	294 (55.5)	2860 (54.3)	3154 (54.4)	
4, 5	153 (28.9)	1607 (30.5)	1760 (30.3)	
*Arrival Day of the Week*, n (%)				<.001^b^
Monday – Thursday	188 (35.6)	2527 (48.0)	2715 (46.9)	
Friday – Sunday	340 (64.4)	2736 (52.0)	3076 (53.1)	
*Time of Arrival*, n (%)				.227
Morning (0700 – 1459)	80 (15.1)	792 (15.0)	872 (15.0)	
Evening (1500 – 2259)	221 (41.7)	2391 (45.4)	2612 (45.0)	
Night (2300 – 0659)	228 (43.1)	2080 (39.5)	2308 (39.8)	

Abbreviations: ED, emergency department; ATS, Australasian Triage Scale; BIBA, brought in by ambulance; BIBP, brought in by police; WI, walked in.

a Event days included public holidays, sporting events, and the day prior and post certain public holidays and sporting events. For further specific dates, see Supplementary Table 3 and Table 4.

b Statistically significant.

“Frequent presenters” to the ED (ie, a person who has six or more ED visits within a 365-day period)^21,22^ were considered, coded, and included in the “other” ARP group. This was because a frequent presenter to the ED is more likely to have a management plan different from that of uncomplicated ARPs, which may influence ED disposition and LOS.^21^

Given the small number of patients who were brought in by police (BIBP) or self-presented/walked in (WI) on event days, the BIBP and WI mode of arrival categories were combined and re-coded as “other” means or mode of arrival for comparison of “uncomplicated” and “other” ARPs.

Of the 11 public holidays in Queensland, prior literature suggests major public holidays are more likely to influence ARPs to ED.^14,18^ Therefore, public holidays included in this study were: Australian and New Zealand Army Corps (ANZAC) Day; Christmas Eve, Christmas Day; Boxing Day; Easter Friday, Saturday, Sunday, and Monday; Labor Day; New Years period; and the Queen’s Birthday.^14,18,23^ Sporting events included: large locally held sporting events - marathon and motorized car racing; state interest sporting events - the State of Origin; and national interest sporting events - the Australian Football League (AFL) Grand Final. These are popular sporting events where celebrations involving alcohol are common.^18^ The day before Australia Day, Labor Day, and the Queen’s Birthday public holiday were included in the “public holiday” day type as increased risk of alcohol consumption has been noted to occur on the day prior to these public holidays.^18,23^ To capture “high alcohol times” that extend into the next day,^24^ the day after a public holiday and sporting event were also included.

To identify the date of the holiday, the type of public holiday and corresponding year were entered into an online search engine on a Queensland Government website which provided specific dates for Queensland public holidays^23^ (Supplementary Table 3; available online only). An online website was used to identify local sporting events occurring between the years 2016 and 2020.^25^ For those sporting events celebrated yet not held locally (State of Origin and AFL Grand Final), online sporting websites were used to obtain past game dates^26,27^ (Supplementary Table 4; available online only).

**Table 3.** Health Service Outcomes of Alcohol-Related Presentations to EDs on Event Days versus Non-Event Days

	Event Day (n = 529)	Non-Event Day (n = 5,252)	Total (n = 5,781)	P Value
**Health Service Outcomes**				
*ED Length of Stay (minutes),* M (IQR)^a^				
All	220 (145-361)	224 (133-366)	224 (134-366)	.730
Admitted	210 (146-317)	224 (153-340)	223 (152-337)	.312
Not Admitted^b^	230 (144-378)	224 (121-386)	225 (123-383)	.307
*Disposition,* n (%)				.834
Admitted	183 (34.6)	1844 (35.1)	2027 (35.0)	
Not Admitted^b^	346 (65.4)	3417 (64.9)	3763 (65.0)	

Abbreviation: ED, emergency department; IQR, interquartile range.

a Analysis excludes patient presentations with an emergency department length of stay ≤0 or > 6000 minutes.

b Not admitted includes disposition of discharged, did not wait, and left after treatment commenced.

**Table 4.** Alcohol-Related Presentations to EDs on Event Days, by Type of Presentation

	Uncomplicated (n = 123)	Other (n = 406)	Total (n = 529)	P Value
**Patient Demographics**				
*Age,* M (IQR)	24 (18-39)	40 (25-51)	36 (22-50)	<.001^a^
*Sex*, n (%)				.842
Male	66 (53.7)	222 (54.7)	288 (54.4)	
Female	57 (46.3)	184 (45.3)	241 (45.6)	
**Presentation Characteristics**				
*Mode of Arrival*, n (%)				.038^a^
BIBA	112 (91.1)	339 (83.5)	451 (85.3)	
Other^b^	11 (8.9)	67 (16.5)	78 (14.7)	
*ATS*, n (%)				.004^a^
1, 2	30 (24.4)	52 (12.8)	82 (15.5)	
3	56 (45.5)	238 (58.6)	294 (55.6)	
4, 5	37 (30.1)	116 (28.6)	153 (28.9)	
*Arrival Day of the Week*, n (%)				.338
Monday – Thursday	39 (32.0)	149 (36.7)	188 (35.6)	
Friday – Sunday	83 (68.0)	257 (63.3)	340 (64.4)	
*Time of Arrival*, n (%)				<.001^a^
Morning (0700 – 1459)	15 (12.2)	65 (16.0)	80 (15.1)	
Evening (1500 – 2259)	37 (30.1)	184 (45.3)	221 (41.8)	
Night (2300 – 0659)	71 (57.7)	157 (38.7)	228 (43.1)	
**Health Service Outcomes**				
*ED Length of Stay (minutes),* M (IQR)				
All	197 (101-301)	226 (157-391)	220 (145-361)	<.001^a^
Admitted	187 (81-292)	218 (157-332)	210 (145-317)	.014^a^
Discharged	213 (107-316)	233 (156-421)	232 (152-390)	.007^a^
*Disposition*, n (%)				.039^a^
Admitted	33 (26.8)	150 (36.9)	183 (34.6)	
Not Admitted^c^	90 (73.2)	256 (63.1)	346 (65.4)	

Abbreviations: ATS, Australasian Triage Scale; BIBA, brought in by ambulance; ED, emergency department; IQR, interquartile range.

a Statistically significant.

b Other includes presentations BIBP (brought in by police) and WI (walked in).

c Not admitted includes disposition of discharged, did not wait, and left after treatment commenced.

### Data Analysis

Descriptive statistics were used to describe the sample, ED presentation characteristics, and outcomes of ARPs to ED on event days and non-event days. As continuous data were not normally distributed, median and interquartile ranges (IQRs) were reported.

To determine if differences existed between ARPs to EDs on event days and non-event days, as well as differences between uncomplicated and other ARPs on event days only, inferential statistics were used. The Pearson’s Chi squared test was used for categorical data and the Mann-Whitney U test was used for continuous data. Statistical significance was set at P <.05. Analyses were undertaken using Statistical Package for Social Sciences, Version 26.0 (IBM Corp.; Armonk, New York USA).

### Ethics

Ethics approval was received from the Health Service (HREC/2020/QGC/61054) and Griffith University (GU Ref No: 2021/310) Human Research Ethics Committees.

## Results

**Figure 1.**
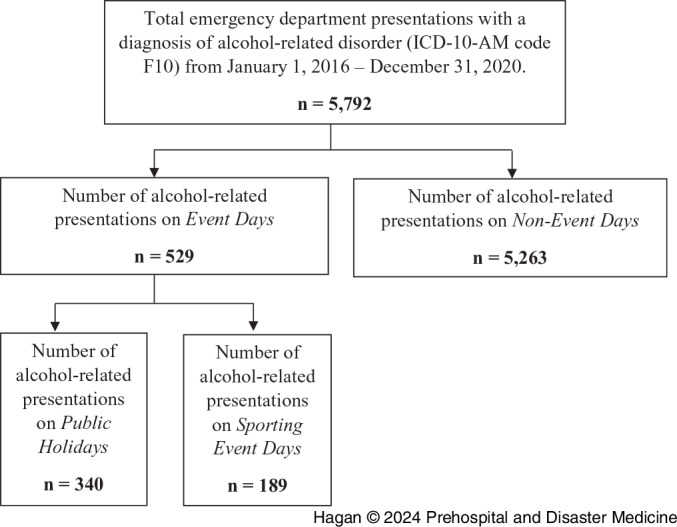
Flow Diagram of Alcohol-Related Presentations to the Emergency Department on Event Days and Non-Event Days. Note: Event days included public holidays, sporting events, and the day prior and post certain public holidays and sporting events. For further specific dates, see Supplementary Table 3 and Table 4. Abbreviation: ICD-10-AM, International Statistical Classification of Diseases and Related Health Problems, Tenth Revision, Australian Modification.

This sample comprised a total of 5,792 ARPs made to the two EDs from January 1, 2016 – December 31, 2020 with a diagnosis of alcohol-related disorder (ICD-10-AM code F10). Of these, 529 (9%) ARPs were on event days and 5,263 (91%) ARPs were on non-event days (Figure 1).

### Patient Demographics

There was no difference in age (median 36 years), sex (55% male), or Aboriginal and/or Torres Strait Islander status (95% not Indigenous) for ARPs on event days versus non-event days (Table 1).

### ED Presentation Characteristics

Statistically significant differences were noted in the type of presentation, mode of arrival, and arrival day of the week when comparing presentation characteristics for ARPs to EDs on event days versus non-event days (Table 2). Regarding type of presentation, a higher proportion of “other” ARPs were evident on both event and non-event days; however, the proportion of “uncomplicated” ARPs was higher on event days than non-event days. For mode of arrival, brought in by ambulance (BIBA) was the most common mode, and this was more evident on event days. For arrival day of the week, a higher proportion of ARPs occurred on Friday-Sunday for both event and non-event days, and this was more pronounced for event days. No difference for triage category and time of arrival was identified.

### Health Service Outcomes

There was no significant difference in the ED LOS or disposition for those presenting with ARPs to EDs on event days versus non-event days. For all ARPs, the median ED LOS was 224 minutes and 35% of ARPs were admitted (Table 3).

### ARPs to EDs on Event Days, by Type of Presentation

The type of ARP was explored in more detail (focusing on event days), given the noted differences in the type of presentation when comparing event day and non-event day. On event days, “uncomplicated” ARPs and “other” ARPs differed for patient demographics (age); presentation characteristics (mode of arrival, ATS, and time of arrival); and health service outcomes (ED LOS and disposition); Table 4. Compared to “other” ARPs, the “uncomplicated” ARPs tended to be younger, comprise a higher proportion of BIBA arrivals, a higher proportion of ATS-1 and ATS-2 presentations, and comprise a higher proportion of night-time (11:00pm-06:59am) arrivals. The ED LOS for “uncomplicated” ARPs was shorter overall and when considered by disposition (admitted or not admitted) compared to “other” ARPs. Furthermore, a smaller proportion of “uncomplicated” ARPs required hospital admission when compared to “other” ARPs. On event days, no significant differences between “uncomplicated” and “other” ARPs were identified for sex or arrival day.

## Discussion

As alcohol consumption continues to be an issue within the Australian community,^28^ progressive efforts to minimize the impact on EDs are needed.^16^ The main finding from this study was that event days had a noteworthy impact on ARPs to EDs. This could be attributed to current laws and legislation surrounding the service and consumption of alcohol. One approach to minimize the impact of ARPs to EDs is through government legislation, examples of which include: (1) the liquor act 1992 which aims to regulate the sale and supply of alcohol within the Australian community to minimize alcohol-related harm and adverse effects;^29^ (2) the introduction of Goods and Services Tax and alcopops tax;^30,31^ and (3) the more recent decriminalization of public drunkenness in Victoria.^28^ Whilst this newest legislation involves a move from a police-led approach to a health-led approach,^28^ the impact on prehospital and ED services is yet to be established.

Alcohol-related presentations mostly arrived to ED by ambulance, more so on event days. Despite this, less than 16% of all ARPs to EDs were considered to have immediately/imminently life-threatening conditions. Combined, this finding reflects the importance of prehospital strategies to reduce pressures on EDs and emergency services. In-event health services (IEHS) at pre-organized events are one such strategy designed to provide on-site health care and early intervention to reduce ambulance transfers to ED.^11^ Whilst this approach aims to reduce ED utilization, IEHSs are not always available at events. Instead, in cases of no IEHS, people often seek assistance from EDs and usually remain there overnight.^4^ Increasing the availability of IEHS is recommended to assist with reducing the impact of ARPs (especially those “uncomplicated” in nature) to EDs on event days.

Along with related Department of Health guidelines (such as the management of patients with acute severe behavioral disturbance in EDs), policy measures, and prehospital strategies, tailored clinical management approaches to sub-sets of ARPs presenting to ED may be of value.^9^ With ARPs being relatively young (median 36 years) and around one in five ARPs considered “uncomplicated,” further efforts to provide a symptomatic management approach for uncomplicated ARPs as common practice^1,10^ is warranted. Although there are currently no formal clinical pathways for the management of an intoxicated patient within the ED, current ED practices of providing supportive symptomatic management through rehydration, checking a blood glucose level, monitoring for signs of withdrawal, and replenishing vitamins such as thiamine have proven to be effective.^1,10^

For more emergent, severe, or complicated ARPs, additional care and emergency resources are understandably required.^9,10,21^ Additional research is recommended to further understand these variations to inform clinical practice, particularly for uncomplicated ARPs and on event days.

## Limitations

There were several limitations within this study. This study was retrospective in nature and limited in generalizability as it was conducted across two EDs. There is possible under-estimation of the true extent of ARPs to EDs due to standard data capture processes and systems (ie, inclusion of primary diagnosis codes only). This study focused on a select number and type of public holidays and sporting events where the consumption of alcohol and binge drinking behaviors are not uncommon.^12,16,18^ The inclusion of other events, day types, or countries with different laws around the consumption of alcohol may have resulted in different findings. An expert panel was used to inform the classification of uncomplicated ARPs. Whilst there may be the possibility of selection bias with this approach, it enabled consistent application to a large volume of data.

## Conclusions

As alcohol consumption continues to be an issue within the Australian community, progressive efforts to minimize the impact on EDs are needed. This study found that public holidays and sporting events had a noteworthy impact on the overall number of ARPs to EDs over the five-year study period. Alcohol-related presentations were heavily reliant on ambulance services for transport to ED, particularly on event days, highlighting the importance of prehospital strategies. With variances noted amongst the type of presentation, alternate care models tailoring clinical management to sub-sets of people presenting to EDs may be of value. Thus, further exploration into the clinical management of uncomplicated ARPs is warranted to inform future resource allocation in general and on event days.

## Supporting information

Hagan et al. supplementary material Hagan et al. supplementary material

## Supplementary Materials

To view supplementary material for this article, please visit https://doi.org/10.1017/S1049023X24000232
