# Supplementary material for: The Impact of Alcohol-Related Presentations to Emergency Departments on Days with a Public Holiday or Sporting Event: A Retrospective Cohort Study
Source: Prehosp Disaster Med. 2024 May 2;39(3):244–50. doi: 10.1017/S1049023X24000232 (PMC11496239; doi:10.1017/S1049023X24000232)
Supplement: Hagan et al. supplementary material [file S1049023X24000232sup001.docx]

**SUPPLEMENTARY TABLES
Supplementary Table 1.** Presenting problems for ‘uncomplicated’ alcohol-related presentations to EDs (for ED presentations diagnosed with alcohol-related disorder (ICD-10-AM code F10)

| **Presenting problem** |
| --- |
| Acute intoxication |
| Altered level of consciousness |
| Altered sensation |
| Collapse |
| Dizziness/vertigo |
| Gait disturbance |
| Nausea/vomiting |
| Unplanned ED presentation |
| Unsettled |

**Supplementary Table 2.** Presenting problems for ‘other’ alcohol-related presentations to emergency departments (for ED presentations diagnosed with alcohol-related disorder (ICD-10-AM code F10)

| **Presenting problem** | | |
| --- | --- | --- |
| Abdo/pelvis/perineal pain | Dyspnoea | Palpitations/abnormal heart rate |
| Abnormal heart rate | Emotional crisis | Pain |
| Abrasion | Environmental temperature | Pedestrian VS |
| Addiction/dependency | Epistaxis | Planned ED recall or overview |
| Aggression | Erythema/rash or  other skin complaint | Post-ictal |
| Agitation | Face-other pain | Psychiatric/behavioural |
| Alleged assault | Fall | Psychosis |
| Altered breathing | Fever/hyperthermia | Regional problems |
| Altered mental state/ neurological symptoms | Fever/hypothermia | Request medication/cert/result/procedure |
| Anxiety/agitation | Gastrointestinal | Requesting investigation |
| Apnoea | GI bleed | Respiratory |
| Back pain | Hallucinations | Review/admissions |
| Behavioural | Head injury | Seizure |
| Behavioural problem | Headache | Self harm |
| Bleeding from | Hyper/hypoglycaemia | Sexual assault |
| Bleeding not associated  with acute injury | Hypertension/hypotension | Skin/skin complaint |
| Blood in urine | Hysterical | Sleep disturbance |
| Blunt injury | Immersion | SOB/IWOB/Wheeze |
| Bruising/other bleeding | Increased respiratory effort/distress | Social concern incl child protection |
| Burn/scald | Injury | Speech disturbance |
| Cardiac | Inpatient team review | Suicidal attempt |
| Cardiac arrest | Intoxication | Suicidal ideation |
| Cardiac vascular | Known mental illness | Suicidal risk |
| Chest pain | Laceration/skin tear | Suicidal-homicidal ideation |
| Confusion/disorientation | Limb/joint pain | Swallowing difficulty |
| Contusion | Mania | Swelling/lump |
| Cough/rhinorrhoea | MB/quadbike-driver | Symptoms signs |
| Crash other vehicle | Mental health | Tachypnoea |
| Crying | Mood disturbance | Tachycardia |
| CVA | Multi-trauma | Temperature stress |
| Cyanosis | MVC driver/passenger | Threatening behaviour |
| Cycle related | Neck/throat pain | Unresponsive |
| Delusions | Neurological | Unplanned ED presentation |
| Dependence/harmful use | Non emergent/reviews | Unsteady |
| Depression | Non-emergent | Urinary symptoms |
| Diabetes/endocrine | Other | Vision loss |
| Diarrhoea | Overdose accidental | Weakness |
| Difficulty walking | Overdose intentional | Weight gain/loss |
| Distended abdomen | Overdose/toxic exposure | Wheeled device |
| Drug/alcohol/poisoning | Paediatric | Withdrawal |
| Dysphasia | Pain not associated with acute injury |  |

Cerebral Vascular Accident (CVA); Emergency Department (ED); Gastrointestinal (GI); Increased Work of Breathing (IWOB);
Motor Bike (MB); Motor Vehicle Car (MVC); Shortness Of Breath (SOB)

**Supplementary Table 3.** Public holidays considered: type and dates

| Type | New Years | Australia Day | Easter | ANZAC day | Labour Day | Queen’s Birthday | Christmas period |
| --- | --- | --- | --- | --- | --- | --- | --- |
| *Year/Dates* |  |  |  |  |  |  |  |
| 2016 | 01/01/16 31/12/16 | 25/01/16† 26/01/16 27/01/16† | 25/03/16 26/03/16  27/03/16  28/03/16 | 25/04/16   26/04/16† | 01/05/16†  02/05/16  03/05/16† | 30/09/2016† 01/10/2016 02/10/16† | 24/12/16  25/12/16  26/12/16  27/12/16† |
| 2017 | 01/01/17  31/12/17 | 25/01/17†  26/01/17  27/01/17† | 14/04/17  15/04/17 16/04/17  17/04/17 | 25/04/17  26/04/17† | 30/04/17†  01/05/17  02/05/17† | 01/10/17†  02/10/17  03/10/17† | 24/12/17  25/12/17  26/12/17  27/12/17† |
| 2018 | 01/01/18  31/12/18 | 25/01/18† 26/01/18  27/01/18† | 30/03/18  31/03/18  01/04/18  02/04/18 | 25/04/18  26/04/18† | 06/05/18†  07/05/18  08/05/18† | 30/09/18†  01/10/18 02/10/18† | 24/12/18  25/12/18  26/12/18  27/12/18† |
| 2019 | 01/01/19  31/12/19 | 25/01/19†  26/01/19  27/01/19† | 19/04/19  20/04/19  21/04/19  22/04/19 | 25/04/19  26/04/19† | 05/05/19†  06/05/19  07/05/19† | 06/10/19†  07/10/19 08/10/19† | 24/12/19  25/12/19  26/12/19  27/12/19† |
| 2020 | 01/01/20  31/12/20 | 25/01/20†  26/01/20  27/01/20† | 10/04/20 11/04/20 12/04/20  13/04/20 | 25/04/20  26/04/20† | 03/05/20†  04/05/20  05/05/20† | 04/10/20†  05/10/20  06/10/20† | 24/12/20  25/12/20  26/12/20  27/12/20† |
| †: day before / after the public holiday Australian and New Zealand Army Corps (ANZAC) | | | | | | | |

**Supplementary Table 4.** Sporting events considered: type and dates

| Type | State of Origin | AFL Grand Final | Gold Coast Marathon | Gold Coast 500 |
| --- | --- | --- | --- | --- |
| *Year/Date’s* |  |  |  |  |
| 2016 | 01/06/16  02/06/16†  22/06/16  23/06/16†  13/07/16  14/07/16† | 01/10/16  02/10/16† | 03/07/16  04/07/16† | 21/10/16  22/10/16  23/10/16  24/10/16† |
| 2017 | 31/05/17  01/06/17†  21/06/17  22/06/17†  12/07/17  13/07/17† | 30/09/17  31/09/17† | 02/07/17  03/07/17† | 20/10/17  21/10/17  22/10/17  23/10/17† |
| 2018 | 06/06/18  07/06/18†  24/06/18  25/06/18†  11/07/18  12/07/18† | 29/09/18  30/09/18† | 01/07/18  02/07/18† | 20/10/18  21/10/18  22/10/18† |
| 2019 | 05/06/19  06/06/19†  23/06/19  24/06/19†  10/07/19  11/07/19† | 28/09/19  29/09/19† | 07/07/19  08/07/19† | 26/10/19  27/10/19  28/10/19† |
| 2020 | 04/11/20  05/11/20†  11/11/20  12/11/20†  18/11/20  19/11/20† | 24/10/20  25/10/20† | 04/07/20  05/07/20  06/07/20† | 30/10/20  31/10/20  01/11/20  02/11/20† |
| †: day before / after the sporting event  Australian Football League (AFL) | | | | |
